# Mother-to-Children *Plasmodium falciparum* Asymptomatic Malaria Transmission at Saint Camille Medical Centre in Ouagadougou, Burkina Faso

**DOI:** 10.1155/2014/390513

**Published:** 2014-11-23

**Authors:** Zoenabo Douamba, Nangnéré Ginette Laure Dao, Théodora Mahoukédé Zohoncon, Cyrille Bisseye, Tegwindé Rebeca Compaoré, Jacques Gilbert Kafando, Bavouma Charles Sombie, Djeneba Ouermi, Florencia W. Djigma, Paul Ouedraogo, Nadine Ghilat, Virginio Pietra, Vittorio Colizzi, Jacques Simpore

**Affiliations:** ^1^Laboratory of Molecular Biology and Genetics (LABIOGENE), University of Ouagadougou, 03 P.O. Box 7021, Ouagadougou 03, Burkina Faso; ^2^Pietro Annigoni Biomolecular Research Centre (CERBA), 01 P.O. Box 364, Ouagadougou 01, Burkina Faso; ^3^University St. Thomas Aquinas of Ouagadougou, 06 P.O. Box 10212, Ouagadougou 06, Burkina Faso; ^4^Saint Camille Medical Centre of Ouagadougou, 01 P.O Box 364, Ouagadougou 01, Burkina Faso; ^5^Laboratory of Molecular and Cellular Biology, University of Science and Technique of Masuku (USTM), P.O. Box 943, Franceville, Gabon; ^6^Department of Biology, UNESCO Chair in Biotechnology, Laboratory of Immunology & Pathology, University of Rome Tor Vergata, Scientific Research Avenue snc, 00143 Rome, Italy

## Abstract

*Background*. Malaria's prevalence during pregnancy varies widely in parts of sub-Saharan Africa, including Burkina Faso. The objective of this study was to evaluate the incidence of mother-to-child malaria transmission during childbirth at St. Camille Medical Centre in the city of Ouagadougou. *Methods*. Two hundred and thirty-eight (238) women and their newborns were included in the study. Women consenting to participate in this study responded to a questionnaire that identified their demographic characteristics. Asymptomatic malaria infection was assessed by rapid detection test Acon (Acon Malaria Pf, San Diego, USA) and by microscopic examination of Giemsa-stained thick and thin smears from peripheral, placental, and umbilical cord blood. Birth weights were recorded and the biological analyses of mothers and newborns' blood were also performed. *Results*. The utilization of long-lasting insecticidal nets (LLINs) and intermittent preventive treatment with sulfadoxine-pyrimethamine (SP) were 86.6% and 84.4%, respectively. The parasitic infection rates of 9.5%, 8.9%, and 2.8% were recorded, respectively, for the peripheral, placental, and umbilical cord blood. Placental infection was strongly associated with the presence of parasites in the maternal peripheral blood and a parasite density of >1000 parasites/*µ*L. *Conclusion*. The prevalence of congenital malaria was reduced but was associated with a high rate of mother-to-child malaria transmission.

## 1. Introduction

In sub-Saharan Africa, about thirty-two million pregnant women are living in areas at risk for* Plasmodium falciparum* malaria transmission [1]. The prevalence of gestational malaria is highly variable in these regions. Indeed, prevalence of 5%, 28%, and 68.3% was reported, respectively, in Ghana [2], Uganda [3], and Nigeria [4]. Two previous studies in Burkina Faso showed that the prevalence of malaria among pregnant women ranged from 28.2% to 42.7% [5, 6]. Malaria infection during pregnancy is potentially dangerous because of the risk for maternal and infantile health [7]. Even asymptomatic,* Plasmodium falciparum* caused malaria is responsible for anemia in pregnant women [6, 8]. The severity of malaria in pregnant women is due to placental sequestration of infected red blood cells [9, 10]. This sequestration alters placental function [11] and causes nutrient deficiencies that result in abortions, intrauterine growth retardation, stillbirth, and premature birth [12, 13]. Gestational malaria is responsible for a high maternal and infantile morbidity and is causing about 75 000 to 200 000 deaths of infants each year [14]. Malaria vertical transmission could have serious consequences for the newborn such as a high susceptibility to malaria infection during the first months of life [15, 16] and also an early susceptibility to other infections [17, 18]. This susceptibility to infection is due to neonatal T cells imbalance and proinflammatory and anti-inflammatory immune responses after their sensitization by* Plasmodium falciparum in utero* [19–21]. Placenta malaria infection is high in Burkina Faso and it is characterized by a low rate of neonatal infection [5]. However, few studies have investigated malaria vertical transmission and its impact on the newborns' health. The objectives of this study were (i) to assess the coverage of malaria prophylaxis with intermittent preventive treatment (IPT) based on sulfadoxine-pyrimethamine (SP) in parturients, (ii) to determine the prevalence of asymptomatic malaria in parturients, and (iii) to estimate the prevalence of placental malaria in asymptomatic pregnant women and the incidence of congenital malaria among their newborns.

## 2. Materials and Methods

### 2.1. Study Population

This cross-sectional study was conducted from September 2013 to February 2014 at Saint Camille Medical Center (CMSC) of Ouagadougou. Ouagadougou is located in the center of Burkina Faso with over a million and a half inhabitants. The climate in the area is characteristic of the Sudanese savannah, with a rainy season from June to October, the cold and dry season from November to January, and the hot and dry season from February to May. The transmission of malaria is stable with a high seasonal transmission from June to November. Pregnant women without clinical signs of malaria and who gave birth were enrolled in this study. Symptoms of clinical suspicion of malaria include fever, headache, vomiting, nausea, abdominal pain, diarrhea, and myalgia. Since malaria symptoms can be confused with clinical manifestations occurring in pregnant women during labor and delivery, only nonfebrile pregnant women were considered asymptomatic for malaria. Only 238 women who consented to participate in the study were recruited. These women completed a questionnaire to determine their demographic characteristics and the malaria prophylaxis used.

### 2.2. Blood Collection

From the women who consented to participate in the study, venous, placental, and umbilical cord blood was collected in an EDTA impregnated tube for hematological tests or dry tube for biochemical analysis.

### 2.3. Biological Analysis

Asymptomatic malaria infection was assessed by rapid detection test (RDT) Acon (Acon Malaria Pf, San Diego, USA) and by microscopic examination of Giemsa-stained thick and thin smears from peripheral, placental, and umbilical cord blood. All slides were read twice by experienced microscopists and the discrepancies resolved by a third reader (limit of detection, approximately 2 parasites/mL). We used the standard method to determine parasitaemia as in most studies conducted in Africa. For thick films, parasites and leukocytes were counted in the same fields until 200 or 500 leukocytes were counted. Parasite densities were estimated using an assumed leukocyte count of 8,000 leukocytes/*μ*L. Parasite densities were calculated according to the following formula: number of parasites counted × 8000/number of leucocytes counted [5, 22]. Haemoglobin level test was performed using a commercial colorimetric kit (Cypress Diagnostics, Langdorp, Belgium) following the manufacturer's instructions. The determination of aspartate aminotransferase (ASAT), alanine aminotransferase (ALAT), and creatinine levels was performed using commercial kits in a Cobas machine (Roche Diagnostics GmbH, Sandhofer Strasse).

### 2.4. Statistical Analysis

Data were analyzed using Statistical Package for Social Sciences (SPSS version 20; SPSS Inc., Chicago, IL, USA) and EPI-Info version 6.04 dfr (CDC, Atlanta, GA, USA). *P* values below 0.05 were considered statistically significant.

## 3. Results

### 3.1. Parturients' Sociodemographic Characteristics

A total of 238 parturients aged from 17 to 42 years were included in the study. The mean age of women was 26.46 ± 4.96 years. The age group of 21 to 25 years old was the most represented. The majority of women (86.6%) used long-lasting insecticidal net (LLIN) for vector control. Most women took at least one dose of sulfadoxine-pyrimethamine (SP) as an intermittent preventive treatment against malaria. Anemia was diagnosed in 18.4% of pregnant women, and 3.4% (8/238) had severe anemia (Table 1).

### 3.2. Clinical Characteristics of the Newborns

Two hundred and thirty-eight (238) mother-infant pairs were included in the study. Newborns had a mean weight of 3157 ± 394 grams (2400–4430 g). A small number of children (1.7%) were less than 2500 g birth weight. The mean of hemoglobin level in newborns rate was 14.9 g/dL. Anemia was present in 10.1% of neonates. Over 90% of the infants included in our study had an elevated creatinine. Transaminases were also measured in newborns and over 84% of them had normal aminotransferase levels (Table 2).

No association was found between the malaria infection in parturients and placental and umbilical cord blood and the clinical parameters of newborns.

### 3.3. Prevalence of Asymptomatic Malaria in Pregnant Women

The parasites were present in the peripheral blood of 8.8% (21/238) of the pregnant women.

*P. falciparum* was present in all cases of infections. The plasmodial mixed infections were found in 6 of the 21 mothers diagnosed positive by RDT. Thick smears revealed the presence of* P. malariae* in 2 mixed infections and* P. ovale* in one case. The geometric mean of parasites in the parturients was 14,477 parasites/*μ*L (118–115073 parasites/*μ*L) (Table 3). The infection rate (12.4%) was higher during the period of September to December compared to the one of January to February (8.2%), but the difference was not significant.

### 3.4. Prevalence of Placental Malaria

The microscopy revealed the presence of parasites in placental blood, 7.1% (17/238) of the specimens tested. RDT also showed the presence of 6 mixed plasmodial infections in placental blood, including 2 cases of* P. malariae*. The geometric mean of the parasitaemia observed was 21,630 parasites/*μ*L (ranged 143–112,396 parasites/*μ*L) (Table 3). The placental infection rate was also higher from the period of September to December (9.0%) compared to the one of January to February (1.6%).

### 3.5. Incidence of Congenital Malaria

We diagnosed 27 maternal and placental malaria and 5 of 238 umbilical cord blood infections. The incidence of congenital malaria was 2.1% and the prevalence of mother-to-child asymptomatic malaria transmission was 18.5%.* Plasmodium falciparum* was the only parasite species present in the umbilical cord blood. The parasite density was low in the cord blood compared to those observed in placental and peripheral blood with a mean of 1444 parasites/*μ*L (231–5102 parasites/*μ*L) (Table 3).

### 3.6. Association between Peripheral, Placental, and Umbilical Cord Malaria Parasites and LLINs and Intermittent Preventive Treatment with SP

As shown in Table 4, pregnancy-associated malaria was not associated with the use of LLINs, neither with intermittent preventive treatment with SP (IPT-SP). Of the 17 positive thick placental blood smears, the majority (11/17) was from women with positive thick peripheral blood smears (Table 5). Positive thick cord blood smears were from women with positive thick peripheral blood smears and those with both peripheral and placental parasitaemia. Among the women with both peripheral and placental malaria parasites, the majority of placental infection (9/11) was observed among women with a mean of parasite density superior (>) to 1000 parasites/*μ*L (Table 5).

## 4. Discussion

In areas with moderate or high* P. falciparum* malaria endemicity, WHO recommends the use of LLINs and intermittent preventive treatment with SP (IPT-SP) for pregnant women in order to prevent malaria and its effects during pregnancy. IPT with at least two doses of sulfadoxine-pyrimethamine administered during the second and third trimester of pregnancy is an alternative prevention strategy whose effectiveness has been demonstrated by several studies in Burkina Faso and elsewhere in sub-Saharan Africa [23, 24] by reducing the rate of maternal and placental* Plasmodium falciparum* infestation. Most women of our study received two doses of SP in the second and third trimester of their pregnancies. Those who received one dose came in their third trimester.

In this study, the use of malaria preventive measures such as LLINs and IPT-SP was high: 86.6% and 84.5%, respectively. We observed an increase rate in the use of LLINs and IPT compared to the rates of 42% and 27% reported in 2012 in the same health center [8]. This could be explained by the positive impact of awareness campaigns among pregnant women on the use of LLINs and their wide distribution in the population by the Ministry of Health. Our results are similar to those observed in 2013 in the two major cities of Burkina Faso [25]. However, this rate is higher than that reported recently in Ghana [2].

The mean birth weight in the present study is comparable to that reported in a previous study in Burkina Faso [5]. We found a prevalence of 1.7% of low birth weight. This prevalence is much lower than that of 15.8% reported by Ouédraogo et al. [5] but is comparable to the prevalence of 3.3% reported recently in Ghana [2]. The prevalence of anemia among newborns was of 10.1%. This rate is lower than those reported in two previous studies in Malawi (23.4%) and Ghana (40%) [26, 27]. This difference could be explained by the high prevalence of anemia among parturients and by the presence of placental and neonatal malaria in their study populations. A parasite infection rate of 8.8% was found in the peripheral blood of pregnant women. It is less than 28% and 68.3%, respectively, reported in Uganda [3] and Nigeria [4]. This difference could be due to the high IPT-SP adherence rate in Burkina Faso [28] and the high prevalence of malaria among pregnant women in some parts of Nigeria [29–31]. Malaria transmission is higher in Burkina Faso, from the period of July to December, during the rainy season. The parasite infection rate in parturients was higher during the period of September to December.

In this study,* Plasmodium malariae* was identified in 11.1% of asymptomatic malaria cases in pregnant women. Our results are consistent with those of a previous study among children which had reported the prevalence of* P. malariae* ranging from 0.9 to 13.2% in Burkina Faso [32]. The prevalence of* P. malariae* reported in this study is higher than the prevalence of 3.6% found in Nigeria in pregnant women [33]. Despite the low prevalence of* P. ovale* in Burkina Faso, which varies from 0.5 to 1.8 [32], we identified 3.7% of* P. ovale* asymptomatic cases in parturients in this study. This is consistent with a recent study which has shown that* P. ovale* and* P. malariae* represent 5.9% of malaria infections in Senegal [34]. The prevalence of placental malaria was 7.1% among the women. This prevalence is lower than the prevalence of 19.5% and 55.2% reported in Burkina Faso [5] and Nigeria [31], respectively. Moreover, the placental malaria prevalence is elevated than that reported in a recent study in Ghana [2]. The difference found could be explained by an intraregional variation in the prevalence of placental and neonatal malaria [5, 30]. The malaria infection rate of umbilical cord blood was 2.1%. This rate is comparable to the rate of 1.4% reported in two previous studies in Burkina Faso [5] and India [35].

There is a strong association between maternal parasite density, placental malaria, and cord blood parasitaemia. This association was also shown in Burkina Faso [5, 36]. However, we did not find an association between maternal and placental malaria infection and umbilical cord parasitaemia. This could be explained by the limited number of cases of congenital malaria in our study.

Our study has some limitations such as the sample size but also the threshold of parasites detection related to standard microscopy. In fact, the latter may underestimate the number of asymptomatic malaria cases among parturients and placental and cord blood malaria.

## 5. Conclusion

The high use of preventive measures such as LLINs and IPT-SP among parturients has resulted in the reduction of asymptomatic malaria and severe anemia. These efforts should be pursued because there is a high risk of mother-to-child asymptomatic malaria transmission.

Asymptomatic malaria in women is underestimated because placental malaria can be observed in parturients in the absence of parasites in their peripheral blood.

## Figures and Tables

**Table 1:** Characteristics of parturients.

Characteristics	Number (%)
Age (years)	
≤20	15 (6.30)
21–25	101 (42.44)
26–30	76 (31.93)
31–39	46 (19.33)
Hemoglobin level (g/dL)	
<7 (severe anaemia)	8 (3.36)
7–9.9 (moderate anaemia)	13 (5.46)
10–10.9 (slight anaemia)	25 (10.50)
≥11 (no anaemia)	192 (80.67)
Number of SP intakes	
0	37 (15.55)
1	55 (23.11)
2	145 (60.92)
3	1 (0.42)
Long-lasting insecticidal net (LLIN)	
Yes	206 (86.55)
No	32 (13.45)

**Table 2:** Characteristics of newborns.

Characteristics	Number (%)
Weight (gram)	
<2500 (low)	4 (1.68)
≥2500 (normal)	234 (98.32)
Hemoglobin level (g/dL)	
<12.5 (anaemia)	24 (10.06)
≥12.5 (no anaemia)	214 (89.92)
Creatinemia (*µ*mol/L)	
53 to 106 (normal)	223 (93.70)
>106 (elevated)	13 (6.30)
Transaminases GOT (U/L)	
<15 (low)	0
15 to 60 (normal)	226 (94.96)
>60 (elevated)	12 (5.04)
Transaminases GPT (U/L)	
<5 (low)	0
5 to 25 (normal)	202 (84.87)
>25 (elevated)	36 (15.13)

**Table 3:** Malaria parasites detected in peripheral, placental, and umbilical cord blood among parturients.

Samples	Thick peripheral blood smears		Thick placental blood smears		Thick cord blood smears	
	Parasite density (parasites/*µ*L)	Type of plasmodium parasite	Parasite density (parasites/*µ*L)	Type of plasmodium parasite	Parasite density (parasites/*µ*L)	Type of plasmodium parasite
M4			143	*P. f. *		
M6	8,157	*P. f. *				
M16	390	*P. f. *				
M38	14,811	*P. f. *	12,256	*P. f. *		
M49	11,647	*P. f. *	12,317	*P. f. *	5,102	*P. f. *
M66	11,647	*P. f. *and *P. m. *	522	*P. f. *	231	*P. f. *
M90			175	*P. f. *		
M92	1,534	*P. f. *			512	*P. f. *
M95	270	*P. f. *	509	*P. f. *and *P. m. *		
M114	13,044	*P. f. *				
M126	118	*P. f. *	716	*P. f. *	517	*P. f. *
M131	135	*P. f. *				
M133	827	*P. f. *				
M147	26,862	*P. f. *	16,196	*P. f. *		
M155	1,450	*P. f. *and *P. m. *	588	*P. f. *and* P. m. *		
M160			2,470	*P. f. *		
M162			2,263	*P. f. *		
M167	115,073	*P. f. *	112,396	*P. f. *		
M169			18,600	*P. f. *		
M173	16,985	*P. f. *	82,325	*P. f. *		
M175	61,635	*P. f. *	99,445	*P. f. *		
M177	4,610	*P. f. *and *P. o. *	1,064	*P. f. *		
M186	2,370	*P. f. *				
M195	2,857	*P. f. *				
M196	2,105	*P. f. *			857	*P. f. *
M199	1,200	*P. f. *				
M200			5,720	*P. f. *		

*P. f.: Plasmodium falciparum; P. m.: Plasmodium malariae; P. o.: Plasmodium ovale*. Parasite densities presented are from *P. falciparum*.

**Table 4:** Association between intermittent preventive treatment and use of LLIN and pregnancy-associated malaria.

Parameters	Thick peripheral blood smears		*P* value
	Negative	Positive	
IPT with SP			
No	34/37 (91.9%)	3/37 (8.1%)	NS
Yes	181/201 (90.1%)	20/201 (9.9%)	
LLINs			
No	28/32 (87.5%)	4/32 (12.5%)	NS
Yes	187/206 (90.8%)	19/206 (9.2%)	

NS: not significant.

**Table 5:** Association between maternal malaria infection and IPT-SP and placental and neonatal malaria.

Parameters	Thick placenta blood smears		*P* value	Thick cord blood films		*P* value
	Negative	Positive		Negative	Positive	
Thick peripheral blood films						
Negative	209/217 96.3%	8/217 3.7%	<0.001	216/217 99.5%	1/217 0.5%	NA
Positive	12/21 57.1%	9/21 42.9%		16/21 76.2%	5/21 23.8%	
IPT-SP						
No	34/37 91.9%	3/37 8.1%	NS	36/37 97.3%	1/37 2.7%	NA
Yes	182/201 90.5%	19/201 9.5%		196/201 97.5%	5/201 2.5%	
Parasite density						
<1000	3/11 27.27%	8/11 72.73%	NS	0/5 0%	1/5 20%	NA
≥1000	7/16 43.8%	9/16 56.2%		0/5 0%	4/5 80%	

NS: not significant; NA: not applicable.
